# Reducing uncertainties about the effects of chemoradiotherapy for cervical cancer: individual patient data meta-analysis*

**DOI:** 10.1590/S1516-31802010000300011

**Published:** 2010-05-06

**Authors:** Wagner José Gonçalves

**Affiliations:** Scientific coordinator, Department of Obstetrics and Gynecology, Associação Paulista de Medicina (APM), São Paulo, Brazil.

Cervical cancer is the second most common cancer among women worldwide and is the main cancer affecting women in sub-Saharan Africa, Central America and south-central Asia. In countries where effective screening programmes have been implemented for some time (North America, parts of Europe, Australia and New Zealand) there has been a significant decline in the incidence of cervical cancer and associated mortality. However, for those women who are diagnosed with cervical cancer that cannot be removed effectively by surgery alone (bulky early disease or locally advanced disease), the mainstay of treatment until 1999 was radical radiotherapy. In that year, the results of five randomised trials led to a National Cancer Institute (NCI) recommendation that concomitant chemoradiotherapy should be considered for women with cervical cancer, and this has since become a standard of care.

A subsequent Cochrane review by John Green and colleagues^1^ concurred with this recommendation, but the results of the review and the authors’ conclusions suggested that a number of important questions might only be addressed by the collection and re-analysis of individual patient data (IPD) from all relevant randomised trials. Furthermore, of the five trials on which the NCI guidance was based, three used additional treatments for the control groups, which made the true effect of chemoradiotherapy compared to the same radiotherapy difficult to assess. There were also other clinical differences between trials and statistical heterogeneity in the review findings.

Therefore, this new Cochrane review was initiated, based on IPD. The researchers found 25 eligible trials that had compared concomitant chemoradiotherapy (with or without surgery) with the same radiotherapy (with or without surgery), as well as three further trials that used additional treatments for the control groups, but had contributed to the NCI guidance. They could not obtain data from 10 trials (including 1,113 patients) either because they were unable to make contact with the investigators, or because the original investigators were unable to locate the data. Data were obtained for 18 trials including 4,818 women. The main analyses were based on the 15 trials which had an unconfounded comparison of chemoradiotherapy versus radiotherapy. The three additional trials that had used different or additional treatment on the control arm but contributed to the 1999 NCI guidance were not eligible for the main analyses, but were included in a separate sensitivity analysis.

Eleven trials used cisplatin based chemoradiotherapy, three used non-cisplatin based regimens and one three-arm trial compared both a cisplatin and a non-cisplatin based treatment arm with a single control group. All used similar radiotherapy schedules, although one trial had not given brachytherapy because chemoradiotherapy was given in addition to upfront radical surgery. Two of the eleven trials of cisplatin-based chemoradiotherapy gave additional chemotherapy after chemoradiotherapy.

For overall survival, the IPD review found a benefit of chemoradiotherapy. There was a significant difference in the size of the benefit according to whether all the chemotherapy was given entirely with radiotherapy (HR = 0.81, 95% CI 0.71 – 0.91, P = 0.0006) or whether additional chemotherapy was given after chemoradiotherapy (HR = 0.46, 95% CI 0.32 – 0.66, P = 0.00002). The subsequent analyses were restricted to the group of trials that gave chemotherapy entirely with radiotherapy (13 trials in total). These found no evidence that the size of the benefit of chemoradiotherapy varied according to the choice of chemotherapy agent used, planned radiotherapy dose or duration, or the chemotherapy dose or cycle length, but the power of these analyses was more limited and the radiotherapy used in all trials was broadly similar.

This same group of 13 trials showed benefits of chemoradiotherapy for disease-free survival, local and distant recurrence-free survival and time to local or distant recurrence, although there was a smaller and less convincing improvement in the time to distant recurrence.

The effect was consistent in patient subgroups defined by age, tumour histology, grade and whether or not pelvic nodes were involved. However, there was the suggestion of variation in the size of the benefit by tumour stage, with smaller benefits for patients with more advanced tumour stage. Unfortunately, there were too few data available on late complications of treatment to support formal analysis.

All data were obtained for the three trials in the sensitivity analysis trials (1,366 women) and this showed a large increase in heterogeneity when these trials were included alongside the 13 trials of the main analysis, such that there was a significant difference in the size of the treatment effect, both for the group of trials using additional hydroxyurea for the control group (test for interaction P = 0.029) and for the single trial that used extended field radiotherapy for the control group only (test for interaction P = 0.004). The reviewers also noted that survival of women in the control group for these 3 trials was lower than that of the main group of 13 trials.

The findings of this review, including data from 18 trials from 11 countries, provide an unconfounded estimate of the effect of chemoradiotherapy compared with radiotherapy for women with cervical cancer. The results endorse the recommendations made by the NCI in 1999 but with far greater reliability and precision regarding the benefits of chemoradiotherapy. The effect of chemoradiotherapy seems to be consistent whether cisplatin-based or non-cisplatin-based chemotherapy is used, and, as such, non-cisplatin-based regimens may be an option for women who are not able to tolerate cisplatin. The benefit of chemoradiotherapy on all outcomes seems consistent for all radiotherapy doses and schedules, and by the cycle length or dose intensity of the chemotherapy employed, such that there is insufficient evidence to suggest any one treatment schedule over another. Furthermore, the benefit is consistent for women of all ages, histology, grade, or pelvic nodal involvement; although this benefit may be less for women with the higher stages of disease.

Finally, these results suggest additional benefit from giving further chemotherapy after chemoradiotherapy, which requires further testing in the context of randomised trials.

## COMMENTS

This is an excelent individual patient data meta-analysis for those women diagnosed with cervical cancer which cannot be removed by surgery.

This review endorses the success of chemoradiotherapy, compared with radiotherapy, for overall survival and disease free-survival among women with cervical cancer.
